# Microplastics Detection in Streaming Tap Water with Raman Spectroscopy

**DOI:** 10.3390/s19081839

**Published:** 2019-04-18

**Authors:** Ann-Kathrin Kniggendorf, Christoph Wetzel, Bernhard Roth

**Affiliations:** 1 Hannover Centre for Optical Technologies, Leibniz University Hannover, Nienburger Str. 17, 30167 Hannover, Germany; christoph.wetzel@hot.uni-hannover.de (C.W.); bernhard.roth@hot.uni-hannover.de (B.R.); 2 Cluster of Excellence PhoenixD, Leibniz University Hannover, Welfengarten 1, 30167 Hannover, Germany

**Keywords:** microplastics, single particle detection, tap water, Raman spectroscopy

## Abstract

Microplastic particles have been found in drinking water sources worldwide and, thus, also in our food and beverages. Especially small microplastics, with sizes of 1 mm and less, cannot be identified reliably without spectroscopic means such as Fourier transform infrared spectroscopy (FTIR) or Raman spectroscopy, usually applied to the particles extracted from the samples. However, for drinking and tap water, with its comparatively low biological loads, direct observation may be possible and allows a point-of-entry monitoring for beverages and food to ensure uncontaminated drinking water is being used. In a proof of concept, we apply Raman spectroscopy to observe individual microplastic particles in tap water with added particulate and fluorescent contaminants streaming with 1 L/h through a custom-made flow cell. We evaluated several tubing materials for compatibility with microplastic suspensions containing three different polymers widely found in microplastic surveys worldwide. The experiment promises the monitoring of streaming tap water and even clear surface waters for microplastics smaller than 0.1 mm.

## 1. Introduction

Microplastics, defined as plastic particles smaller than 5 mm but larger than 0.1 µm, originate directly as microbeads from personal care products and production pellets during the fabrication of larger plastic parts [1,2], and indirectly as microfibers from synthetic cloth during the washing process and the fragmentation of larger plastic debris such as packaging and car tires, among others [1,3,4,5,6]. It has been found on land and in water bodies worldwide including karst groundwaters and other drinking water sources, as well as in our foods and beverages [5,6,7,8,9,10,11,12].

Analyzing the microplastic content of water usually involves sampling and sample processing before identification and quantification of the microplastic content of a given sample. For waterborne microplastics in smaller volumes or controlled outlets such as water pipes, the sampling is often done with filters and sieves. The detectable particle size is thus determined by the mesh size of the used filters. The filtrate is then further treated to remove organic components before analyzing the remaining particles for polymer content. A succinct overview of the sampling and filter protocols and the subsequent sample treatments used for microplastics can be found in Ivleva et al. [6].

Sample contamination has been identified as one of the most critical issues for reliable microplastics analysis, requiring extensive preparations such as precision filtering of all involved chemicals, blank controls for each step, and handling of the samples only under clean air conditions [6,10,12,13,14]. For example, Mintenig et al. [12] screened large volumes of drinking water derived from groundwater wells at several processing steps from raw water to the household tap, filtering between 300 L (raw water) and 2500 L (tap water) through 3 µm filter units rinsed with analytical grade water and opened only inside the lab. Blank samples consisted of 150 L of 3 µm filtered drinking water. Particles identified as polyethylene (PE), polyamide (PA), polyester (PES), polyvinylchloride (PVC), and epoxy resin sized between 50 and 150 µm were found in low concentrations between 0 and 7 microplastic particles per m^3^ water. However, fibers were found in all their samples, including the blank controls, further stressing the requirement of handling samples only under clean air conditions to avoid likely airborne sample contamination [12]. Therefore, the number of sampling and processing steps occurring before identifying the microplastics within the sample should be as small as possible.

The reference methods for the reliable identification of microplastics among other particles to date are Raman (micro)-spectroscopy and FTIR-spectroscopy [6,15,16,17], though recently also more destructive thermoanalytical methods [14,18] and staining of either the biological content (Rose-Bengal [19]) or the polymers directly (Nile Red [20,21]) have been successfully used for the detection of small (50–500 µm) and very small (0.1–50 µm) microplastics. For example, Mason et al. [11] screened 11 brands of packaged water purchased globally for microplastic contamination using a combination of Nile Red tagging and subsequent FTIR spectroscopy for confirmation.

FTIR- and Raman micro-spectroscopy have been evaluated extensively for their suitability for microplastics analysis because both techniques are non-invasive and can be applied directly on the filter holding the extracted particles [6]. For example, Käppler et al. [15] compared attenuated total reflection (ATR) FTIR-imaging and Raman imaging with a standard charge-coupled device (CCD) camera detection and 532 nm excitation by applying both methods to microplastics extracted from marine sediment samples directly on the silicon filters (hole size 10 µm, filter area 64 mm^2^) used to concentrate the purified particles, recommending FTIR imaging for particles sized 50–500 µm, and Raman imaging for particles between 1 and 50 µm, mostly because ATR-FTIR imaging was faster and equally reliable for the larger particle set. While Cabernard et al. [17] compared FTIR imaging in reflection mode and automated single-particle exploration connected with Raman micro-spectroscopy with excitation at 785 nm for microplastics with sizes between 50 and 500 µm on gold-coated polycarbonate filters, reporting higher particle identification rates for Raman spectroscopy in all particle size classes. However, they had to operate with a reduced laser intensity of only 5–7 mW and, thus, integration times of 30 s were required to avoid damaging the filter material holding the particles.

FTIR- and Raman micro-spectroscopy have been used in many studies and surveys for microplastics. An overview of works reported, including categorization for particle sizes and applied identification method, can be found in Ivleva et al. [6]. More recently, Oßmann et al. [5] used micro-Raman spectroscopy at 532 nm on very small particles from packaged water caught on an aluminum-coated polycarbonate membrane filter with a pore size of 0.4 µm, while Schymanski et al. [10] screened drinking water in reusable and single-use plastic bottles using micro-Raman spectroscopy at 532 nm on gold-coated polycarbonate filters with a sampling volume of 100 mL per sample.

However, Raman spectroscopy is one of the few techniques that can be readily applied to aquatic samples directly [22], thus avoiding many of the challenges that occur when analyzing dried material on sensitive filter surfaces, thus for drinking and tap water, with its comparatively low biological loads, direct observation of microplastic particles is an option. Moreover, all the survey techniques reported to date do not offer the opportunity to investigate microplastics contamination continuously over time. For example, to the best of our knowledge, there is no study on how microplastics contamination correlates with stagnant, high, or low water use in pipe systems, although Mintenig et al. concluded that the majority of the microplastics found in tap water originate from the groundwater processing and the pipe systems [12].

In this work, we present the proof of concept for a straightforward detection based on Raman spectroscopy for individual small microplastic particles of sizes near 0.1 mm in tap water streaming with 1 L/h. The setup can be attached directly to a tap without the need for additional sampling or sample processing, allowing the monitoring of the water for microplastics and other particulates over time. The setup was tested with microplastics of five common polymers against a background of autofluorescence and other particulate contaminants of similar size.

## 2. Materials and Methods

### 2.1. Microplastics, Chemicals, and Prepared Sample Suspensions

Microplastic samples used throughout the experiment were purchased in dry form from Carl Roth GmbH, Karlsruhe, Germany (PA), Sigma-Aldrich Chemie GmbH, Taufkirchen, Germany (PE, PMMA), and Polysciences Europe GmbH, Hirschberg, Germany (PP, PS). Polymer composition, shapes, and sizes of the microplastic particles were confirmed using a confocal Raman microscope (CRM 200 by WITec, Ulm, Germany). Table 1 shows the physical properties of the respective particles and the Raman lines used for identifying the respective polymers in this work. Brij^®^ L4 surfactant, laboratory grade humic acid, and glass microbeads with sizes ranging from 212–300 µm and sizes from 106 µm and smaller were purchased from Sigma-Aldrich Chemie GmbH, Taufkirchen, Germany.

Test suspensions were created with 1 g of the respective microplastic in 1 L of purified water. Test suspensions were stored in glass stoppered glass flasks at room temperature for 24 h before use.

Sample suspensions were created with 0.5 g of microplastic and the respective contaminant in 1 L of tap water drawn at Nienburger Straße 17, 30167 Hannover, Germany. Water contaminants used were 50 mL/L surfactant (Brij^®^ L4) or 10 g/L humic acid. In total, 5 g of microglass was used as particulate contaminant. Sample suspensions were stored in glass stoppered glass flasks at room temperature for 24 h before use.

Microplastic volumes were chosen for a time-efficient test of the setup, paying attention to overall operation time and the risk of particle agglomeration or multiple particles passing the flow cell simultaneously.

Particles were forced into the water column by vigorous shaking of the closed flask and kept in the water column for the duration of the experiment by magnetic stirring.

### 2.2. Water Circuit Setup, Microplastics Compatibility Tests, and Cleaning Protocol

The flow cell was formed by a custom-made rectangular tube of Borosilicate glass (4 × 4 × 50 mm^3^) with a wall thickness of 0.5 mm. Tubing options (Rotilabo^®^ polytetrafluoroethylene (PTFE), Rotilabo^®^ PVC, and Tygon^®^ 2375) with an inner diameter of 5 mm were purchased from Carl Roth GmbH, Karlsruhe, Germany. PTFE tubing was used in the final setup since these tubes showed the least number of adhering particles for all tested microplastics (see Section 3.1). The tubing was directly attached to the flow cell and sealed against leakage with Parafilm^®^ M by Bemis Company Inc., Neenah, WI, USA.

Component compatibility with microplastics was tested by pumping 1 L of each test suspension through the components at a flow rate of 1 L/h, before draining and drying the components, and microscopically analyzing 5 cm^2^ of the surfaces in contact with the test suspensions for attached microplastics. Tubes were kept in a vertical coil during pumping and the analyzed areas were evenly spread throughout the tube length.

A programmable peristaltic pump (iPump 2F by Landgraf Laborsysteme HLL GmbH, Langenhagen, Germany) equipped with a six-roller head and PTFE-coated pump tubes was used to generate a flow of 1 L/h through the flow cell. Effects of the pump tubing and the tube connectors required to link the setup tubing with the pump tubes were minimized by using the pump to pull rather than push the particle suspensions through the flow cell, thus relegating all possible effects of the pump system needed only for the tests to the effluent.

In order to collect primarily individual particles, the tube used to draw the particles through tubing and setup was positioned to avoid contact with glass surfaces or the stirring vortex where agglomeration of the particles was most likely.

The cleaning protocol for the water circuit between experiments consisted of pumping 70% ethanol solution through the circuit for 30 min to remove microbial lifeforms, rinsing with purified water, pumping 1 L of 5% Brij L4^®^ (surfactant) solution through the circuit to remove adhering particles as best as possible, and rinsing again to remove the surfactant.

### 2.3. Opto-Mechanical Setup, Detection Parameters, and Data Procession

The opto-mechanical setup was built on a 300 × 450 mm^2^ base plate and shielded on all sides by 5 mm aluminum plates with drilled openings for the laser fiber, the water tubes, and a USB endoscope camera for monitoring the setup during operation.

**Figure 1:**
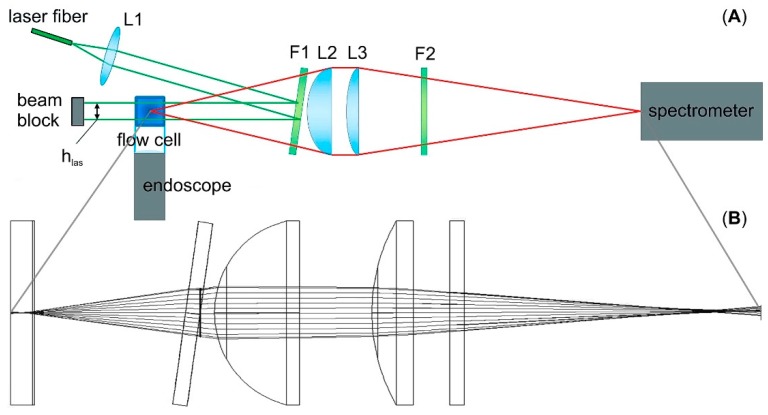
Schematic of the optical setup with orientation of the flow cell perpendicular to the image plane (**A**) and beam path from the center of the flow cell to the entrance slit of the spectrometer (**B**).

The optical setup is shown in Figure 1A. A fiber-connected continuous-wave Nd:YAG laser (RLTMLL-532-3W-5 by Roithner Lasertechnik GmbH, Wien, Österreich) at 532 nm was used for excitation. The beam exiting the laser fiber was collimated to a 1 mm beam width by lens L1 (focal length 8 mm; ACL12708U-A by Thorlabs GmbH, Dachau, Germany) and directed by reflection at an 8° angle on the edge filter (F1) for 532 nm (LP-03-532RU-25 by Semrock Inc., Rochester, MN, USA) through the flow cell. The backscattered light passed through the same filter (F1) and was collected by the lens set L2 (focal length 25.4 mm; LA1951-A by Thorlabs) and L3 (focal length 50 mm; LA113-A by Thorlabs) through a second edge filter F2 (LP-03-532RU-25 by Semrock Inc., Rochester, MN, USA) into a Raman spectrometer (AvaSpec-ULS2048L-USB2 by Avantes Inc., Apeldoorn, The Netherlands). The spectrometer was equipped with a 50 µm slit and a 1200 lines/mm grating blazed for 700 nm with a spectral resolution of 0.61 nm, recording spectra between 200 and 4000 rel. cm^−1^, and an average quantum efficiency of 64% between 625.1 and 641.1 nm, the range containing the dominant Raman lines of most polymers (corresponding to 2800–3200 rel. cm^−1^ at 532 nm excitation). The quantum efficiency of the inbuilt camera at the wavelength range was 27%. The spectrometer had a signal-to-noise ratio (SNR) of 300:1 and a readout rate of 1.8 ms/scan. In order to confirm that the spectra correspond to individual particles and not multiple particles passing through the laser beam simultaneously, the beam path through the flow cell was observed with a USB endoscope camera (Depstech NTC 50HD/70HD Endoscope, Shenzhen Deepsea Yuanhang Investment Co., Ltd., Shenzhen, China), using the flashes of elastically scattered light to determine the number of particles within the beam path during the integration time for each spectrum.

The beam path from the flow cell to the spectrometer as simulated by WinLens3D (Qioptiq Photonics GmbH & Co. KG, Göttingen, Germany) is given in Figure 1B.

For detecting single particles in a streaming medium, the optimal integration time of the spectrometer *T_opt_* depends on the particle speed *v_flow_* with which the particles pass through the laser beam, generating an effective excitation time *T_eff_* for the Raman spectrum determined by the cross-sectional area of the flow cell *A_cell_*, the height of the laser beam *h_las_*, and the pump rate *Q_pump_*:

(1) $$T_{opt}≧T_{eff}=\frac{h_{las}}{v_{flow}}=\frac{h_{las}\cdot A_{cell}}{Q_{pump}}.$$

*T_eff_* = 33 ms for a pump rate of 1 L/h, a 3 × 3 mm^2^ inner cross-section of the flow cell, and a beam width of 1 mm within the flow cell.

Raman spectra covering the whole spectral range of the spectrometer were recorded continuously using Avasoft 8 by Avantes Inc., which also operates the spectrometer. Polymer identification was limited to the spectral range between 2800 and 3100 rel. cm^−1^ to allow for automated identification within the recording time of the next spectrum. Subsequent data processing for presentation was done with QtiPlot [23]. Background removal was done by averaging 500 Raman spectra of flowing tap water without added contaminants and subtracting this averaged background from the recorded Raman spectra. The background-corrected Raman spectra were then smoothed using the Savitsky–Golay filter of QtiPlot.

## 3. Results and Discussion

### 3.1. Microplastics Compatibility

Three types of common laboratory tubing made of polytetrafluoroethylene (PTFE; Teflon^®^), soft polyvinyl chloride (PVC), or thermoplastic olefin (TPO; Tygon^®^) were tested for compatibility with microplastic suspensions. Table 2 gives the number of particles adhering to 1 cm^2^ of the inner tubing surface after 1 L of PA, PE, or PMMA suspension was pumped through.

**Figure 2:**
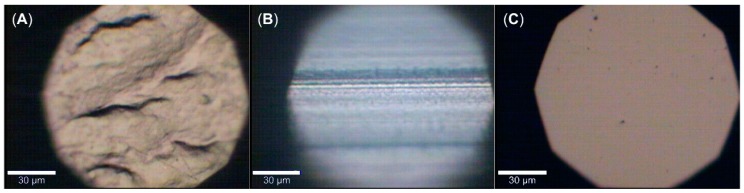
Micrographs of the inner surface of (**A**) polyvinylchloride (PVC), (**B**) polytetrafluoroethylene (PTFE), and (**C**) thermoplastic olefin (TPO) tubing.

Surprisingly, the tubes made of soft PVC and high-density TPO, renowned for their resistance and compatibility to a broad range of chemicals and applications, were found unsuitable for transporting microplastic suspensions [24]. In the case of TPO, the PE and PMMA particles remained attached to the tubing wall even after rinsing the tubes with 0.001 mol/L sulfuric acid (pH 2.75) for two hours. Moreover, the microplastic adherence to the various tubes is dependent on the involved polymers of both tubing and microplastics rather than the particle shape, thus causing the tubes to act as a filter for the respective polymers, resulting in inadvertent underreporting of the affected microplastics. However, we assume the high particle counts in the PVC tubing to be at least partially due to the rough inner surface of these tubes when compared to the inner surfaces of either PTFE or TPO tubing (compare Figure 2).

PTFE tubing was used in the final setup with the caveat that it likely acted as a filter for PTFE particles. However, PTFE is the only major polymer without Raman bands in the observed spectral region between 2800 and 3100 rel. cm^−1^ and it was not found in most of the surveys for microplastics conducted worldwide, which identified the majority of particles as polyethylene terephthalate (PET), PA, PE, PES, PP, PS, and PMMA in varying proportions depending on survey location and procedure [3,5,6,8,9,10].

The glass flow cell was found compatible with all microplastic suspensions, showing no adherent microplastics throughout the measurements. However, particles in contact with the walls of the flow cell decelerated compared to particles passing the flow cell without wall contact.

### 3.2. Microplastics Detection

**Figure 3:**
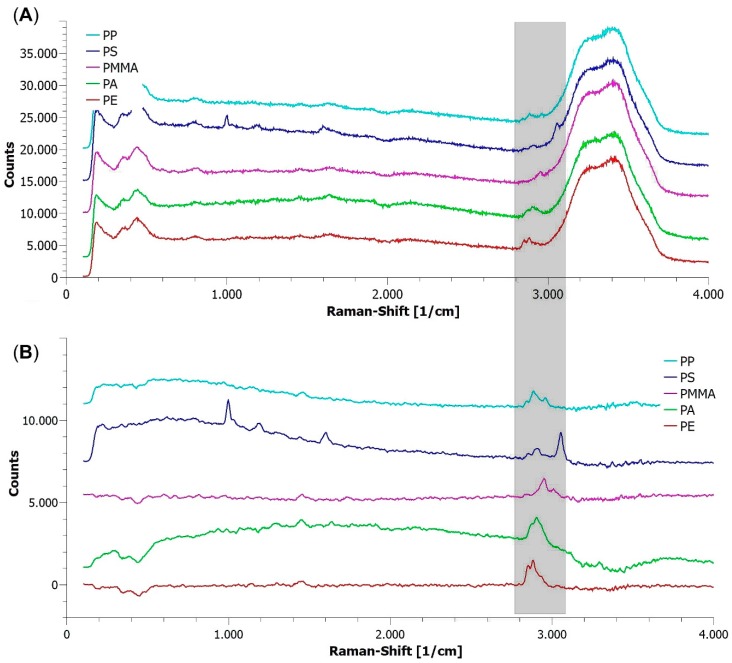
Raman spectra of individual microplastic particles recorded in tap water streaming with 1 L/h through the flow cell: (**A**) as recorded; (**B**) after background removal and smoothing. The shaded spectral range contains the Raman lines used for identifying polymers. Integration time *T_opt_* = *T_eff_* = 33 ms; beam width within the flow cell: 1 mm; laser power within the flow cell: 1.54 W/mm^2^. The spectra were stacked for visibility.

For evaluating the optical setup, microplastic suspended in uncontaminated tap water as described in Section 2.1 was pumped with a flow rate of 1 L/h through the flow cell, resulting in an average effective excitation time *T_eff_* of 33 ms according to Equation (1). The beam width was set to 1 mm to avoid the repeated detection of microparticles decelerating along the walls of the flow cell. Laser power was set to 1.54 W/mm^2^ within the flow cell. The Raman spectra of single microplastic particles streaming through the flow cell are given as recorded in Figure 3A and after data processing, as described in Section 2.3, in Figure 3B.

As can be seen, individual particles of all five types of microplastics in streaming tap water can be identified with a minimal signal-to-noise ratio (SNR) of 5 for identification using their Raman lines between 2800 and 3100 rel. cm^−1^ even in the raw data. However, the Raman lines at the highest wavenumbers—3058 rel. cm^−1^ for PS, 3002 rel. cm^−1^ for PMMA, and 2928 rel. cm^−1^ for PA—are mounted on the broad Raman band between 3200 and 3600 rel. cm^−1^ belonging to the OH stretching of water (Figure 3A). Removing the water-associated background and smoothing the spectra as described in Section 2.3 improves the SNR to 10, allowing a positive identification of microplastics even in contaminated waters.

**Figure 4:**
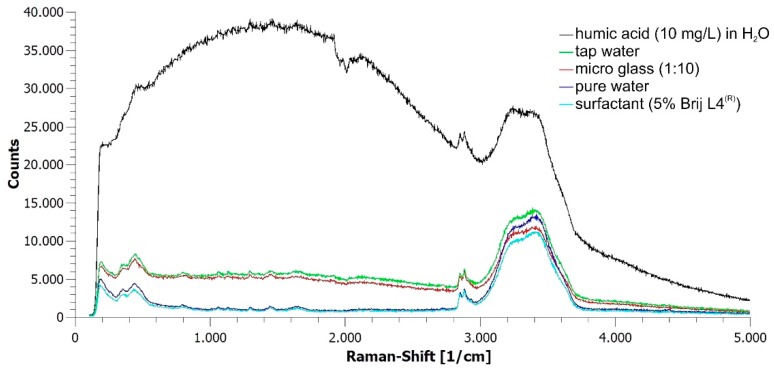
Raman spectra of individual polyethylene (PE) microparticles in water with various contaminants streaming with 1 L/h through the flow cell as recorded. Integration time *T_opt_* = *T_eff_* = 33 ms (16 ms in case of humic acid contamination; beam width within the flow cell: 1 mm; laser power within the flow cell: 1.54 W/mm^2^).

Figure 4 shows the Raman spectra as recorded of individual PE fragments suspended in contaminated tap water streaming with 1 L/h through the flow cell. Contaminants were 5 g microglass, 10 mg/L humic acid, and 5% surfactant. The Raman spectrum recorded of single PE particles in streaming pure water is given for comparison. Excitation time was *T_eff_* = 33 ms except for the sample suspension containing 10 mg/L humic acid, the autofluorescence of which required a reduction of the excitation time to 16 ms to avoid saturation of the spectrometer.

As can be seen, the two Raman bands of PE at 2850 and 2884 rel. cm^−1^ are clearly visible in the spectra of all detected particles, including the Raman spectrum recorded with an excitation time of 0.5 *T_eff_* due to the autofluorescence of the humic acid which caused a noticeable red coloring of the water. Interestingly, the background of the Raman spectrum recorded from a particle in tap water with 5% surfactant is very close to the background seen in the spectrum of a particle in pure water.

The SNR of 5 in the recorded data allows us to clearly separate the polymer Raman lines from those of common biological contaminants like bacteria and microalgae [25,26,27]. While these microorganisms do have Raman lines in the spectral region used for identifying polymers, originating primarily from membranes and proteins within the cells [22,28], they are far weaker compared to the polymer Raman bands originating from the whole particle. In addition, the cell sizes of microalgae (~10 µm) and planktonic bacteria reported in drinking water (~1 µm) are well below the targeted microplastics of 100 µm, further resulting in a very weak contribution of microbial water content to the recorded Raman spectra. In previous works, we studied microbial biofilms at cell level, including incorporated microparticles, finding the polymer Raman spectra to always be much more intense than those of the surrounding microbes of similar size (~1–2 µm) [22,28]. We intend to address this more closely in future works, in which we plan to decrease the detected particle sizes to focus on very small microplastics considered environmentally more problematic plastic pollutants because these readily enter the food chain.

However, microbial aggregates may occasionally have sizes of 100 µm and more, and thus will be detected and seen as a non-polymer particle. Future work will address this task to identify the effects of thick microbial growth covering the microplastics completely, thus screening the polymers. However, growing biofilms of sufficient thickness on microplastics in drinking water is a slow process [25] so that this experiment is time-consuming. Another limiting aspect is the sensitivity to autofluorescence which may saturate the detector at the set integration time, thus blinding the setup temporarily. While we tested the effect of broad fluorescence in the medium itself by adding humic acid to the water, thus mimicking the effect of autofluorescent single cells with sizes below the detection limit in the water, the possibility of autofluorescent microbial aggregates remains.

We do expect the occurrence of these autofluorescent microbial aggregates to be a rare event within properly maintained drinking water systems, though. Nevertheless, work is currently underway to account for these events during the automated data analysis.

To the best of our knowledge, this is the first time that an analysis of microplastics was attempted in streaming water suitable for continuous monitoring of drinking water at a point-of-entry.

For comparison, Mintenig et al. [12] screened 2500 L of tap water derived from groundwater wells, finding microparticles sized between 50 and 150 µm identified as PE, PA, PES, PVC, and epoxy resin in concentrations between 0 and 7 particles per m^3^ water using FTIR-micro-spectroscopy on a 0.2 µm aluminum-coated filter.

Similar research was done on packaged water by Oßmann et al. [5], who analyzed 32 samples of mineral water in plastic or glass bottles bought in Bavaria, Germany, for contamination with very small microplastics (below 10 µm) using micro-Raman spectroscopy at 532 nm on particles caught on an aluminum-coated polycarbonate membrane filter with a pore size of 0.4 µm. Polymers detected in 250 mL of each sample included PET, primarily in PET bottles, while the polymers PE and PP were more often seen in glass bottles than in PET bottles. Styrene-butadiene-copolymer was only found in glass bottles. The authors concluded that most of the microplastic contaminants originate from the cleaning and refilling processes as well as the packaging. However, the sample preparation was extensive, requiring the chemical removal of calcium and magnesium carbonate from the samples, before adding a surfactant to achieve better sample homogeneity, before filtering and subsequently analyzing 4.4% of the filter surface due to the time-consuming particle search. Information about the reusability of the filters were not provided.

Schymanski et al. [10] also screened packaged drinking water in reusable and single-use plastic bottles as well as glass bottles and beverage cartons from grocery stores in Germany for small (50–500 µm) and very small (1–50 µm) microplastics, also using micro-Raman spectroscopy at 532 nm on gold-coated polycarbonate filters with a sampling volume of 100 mL per sample. The polymers found included PET, polyester (PES), PE, PP, and PA among non-polymer particles (i.e., cellulose), likely originating from the packaging.

Finally, a more invasive approach was taken by Mason et al. [11], who screened 11 brands of packaged water purchased globally for microplastic contamination using a combination of Nile Red tagging and subsequent FTIR spectroscopy, reporting an average microplastic density of 325 particles/L, with PP being the prevalent polymer (54%) followed by PA (16%), PS (11%), and PE (10%), with 95% of the detected particles being smaller than 100 µm. Fragment morphology and chemical contaminants within the particles indicated that the plastic contamination, again, originated from the packaging process.

## 4. Conclusions

In this proof of concept, individual particles of small microplastics with particle sizes near 0.1 mm were successfully detected and analyzed in tap water streaming with 1 L/h through a 3 × 3 mm^2^ flow cell against a background of fluorescence and other particles of similar size using Raman spectroscopy with 532 nm laser excitation. The microplastics consisted of five different polymers (polyamide, polyethylene, polymethyl-methacrylate, polystyrene, and polypropylene), either as microbeads or as fragments.

Working with microplastic suspensions requires careful selection of working materials as microplastics easily attach strongly to many polymers commonly used in experimentation.

## Figures and Tables

**Table 1:** Microplastic sample properties.

Abbrev.	Polymer	Particle Sizes [µm]	Particle Shape	Density [g/cm^3^]	Raman Bands ^1^ [rel. cm^−1^]
PA	polyamide	1–315	fragments	1.14	2875, 2903, 2928
PE	polyethylene	1–315	fragments	0.92	2850, 2884
PMMA	polymethyl-methacrylate	15–150	microbead	1.18	2848, 2955, 3002
PP	polypropylene	150	microbead	0.91	2842, 2886, 2961
PS	polystyrene	106–125	microbead	1.05	2855, 2907, 3058

^1^ used for identifying the polymer in the spectral region 2800–3100 rel. cm^−1^; additional bands exist in the traditional fingerprint region below 1800 rel. cm^−1^.

**Table 2:** Number of adhering microplastic particles sized between 1 and 300 µm on 1 cm^2^ of the inner tube surface.

Tubing Material	PA Fragments	PE Fragments	PMMA Microbeads
PTFE	<20	<20	0
PVC ^1^	>1000	>1000	>1000
TPO ^1^	50	>1000	>1000

^1^ particle counting was stopped after a total of 1000 particles was reached.
